# An Adaboost-Backpropagation Neural Network for Automated Image Sentiment Classification

**DOI:** 10.1155/2014/364649

**Published:** 2014-08-04

**Authors:** Jianfang Cao, Junjie Chen, Haifang Li

**Affiliations:** ^1^School of Computer Science & Technology, Taiyuan University of Technology, Taiyuan 030024, China; ^2^Department of Computer Science & Technology, Xinzhou Teachers' University, No. 10 Heping West Street, Xinzhou 034000, China

## Abstract

The development of multimedia technology and the popularisation of image capture devices have resulted in the rapid growth of digital images. The reliance on advanced technology to extract and automatically classify the emotional semantics implicit in images has become a critical problem. We proposed an emotional semantic classification method for images based on the Adaboost-backpropagation (BP) neural network, using natural scenery images as examples. We described image emotions using the Ortony, Clore, and Collins emotion model and constructed a strong classifier by integrating 15 outputs of a BP neural network based on the Adaboost algorithm. The objective of the study was to improve the efficiency of emotional image classification. Using 600 natural scenery images downloaded from the Baidu photo channel to train and test the model, our experiments achieved results superior to the results obtained using the BP neural network method. The accuracy rate increased by approximately 15% compared with the method previously reported in the literature. The proposed method provides a foundation for the development of additional automatic sentiment image classification methods and demonstrates practical value.

## 1. Introduction

With the recent development of multimedia technology and the rapid popularisation of various image capture devices, the scale of digital imagery is expanding rapidly. Numerous gigabyte- (GB-) sized or even terabyte- (TB-) sized digital images are produced, distributed, and shared daily. Confronted with a significant abundance of image data, customers find it increasingly difficult to locate and organise desired images. Therefore, the efficient classification and retrieval of images have attracted considerable attention. Content-based image retrieval (CBIR) is an image retrieval method based on low-level visual features, such as colour, texture, and shape. However, CBIR fails to produce satisfactory retrieval results because it does not completely understand images. CBIR technology only retrieves images from a database on the basis of low-level visual features (colour, texture, and shape), which are not the users' desired retrieval requirements. Images contain vast amounts of emotional semantic information. In practice, customers primarily evaluate the satisfaction of an image based on image-implicated high-level semantic information rather than on low-level features [1]. A semantic gap refers to the distance between low-level and high-level retrieval needs due to inconsistency between the image visual information acquired by the computer and the image semantic information understood by the user. The improvement of low-level visual features and the realisation of high-level semantic-based image retrieval have become increasingly important. The emotional semantic classification of images as a newly emerging technique has become an intense research topic. This technique is capable of retrieving image-emotion semantic adjectives, extracting connotative semantic information, and reflecting human affection towards the image, which constitutes an essential part of the study of digital image comprehension. These characteristics will also enable an examination of multimedia information retrieval and the filtering and interception of illegal information.

Since the late 1990s, numerous studies on image emotion semantic analysis have been conducted. Scholars have focused on the sentiment classification of various types of image data and achieved positive outcomes. Mao et al. proposed an image fluctuation analytical method based on a two-dimensional mathematical wave model and proved that images that adhere to the law of 1/*f* fluctuations produce harmonious and aesthetic perceptions [2]. Li et al. proposed a colour feature-based classification method for house-design images and constructed a relational model between image colour and emotion semantics based on human colour perception and comprehension; a radial basis network classifier was subsequently used to create style classifications for house-design images [3]. Inspired by the psychological idea of “dimension,” Wang et al. constructed an emotion space using semantic quantification and factor analysis and analysed the similarity measurement in the emotion space [4]. They extracted image colour and form features as the perceptual features of the image and mapped the feature space onto the emotion space, using a radial basis function neural network, to create a perception of the image in the emotion space. Li et al. constructed the damping functions of mood and emotions by investigating the relations amongst characters using mood and emotional damping [5]. Based on the emotion-interaction data obtained during the fluctuation of the emotional state, they constructed a multilayer emotion model. Cho and Lee extracted image features using a wavelet transformation and subsequently formulated the retrieval of emotion images based on interactive genetic algorithms [6]. Liu et al. developed an emotion classification of weather images using a support vector machine [7]. Liu et al. proposed a local feature-based semantic clustering method that significantly reduced the scope of image retrieval and improved the efficiency of the method [8]. Shin et al. proposed a method for automatic textile image annotation by predicting emotional concepts from visual features, which can be effectively applied to the commercial textile industry for image retrieval [1].

However, the majority of these studies have focused on the construction of emotional expression vocabularies and the mapping relation between these vocabularies and low-level visual image features. They have typically adopted only one machine-learning method for classification, such as neural networks or a support vector machine, which results in a low classification accuracy.

In “*The Cognitive Structure of Emotions*,” Ortony et al. proposed a cognitive emotion evaluation model known as the Ortony, Clore, and Collins (OCC) model [9]. This model was the first structuralised model applied to the field of artificial intelligence. It provides an emotion classification scheme that benefits from the implementation of and extensive application to computers. This model defines 22 basic emotions and their hierarchical relationships. It represents emotions using a consistent cognitive structure rather than using a psychological perspective [10].

A backpropagation (BP) neural network is a type of multilayer feedforward neural network. Its main characteristics include the forward transfer of signals and the backpropagation of errors. Although this network has been applied to classification, it exhibits the following two distinct drawbacks: it tends to lapse into the local minimum and possesses a slow rate of convergence, which causes it to fail to achieve ideal results when used to solve problems with small sample sizes and high levels of noise. The Adaboost algorithm can improve the classification accuracy of randomly assigned weak classifiers. For this reason, it has been successfully utilised to solve the learning problems encountered by numerous machines.

In this study, we constructed an emotion space using the OCC emotion models. We also utilised the BP neural network as a weak classifier and integrated its multiple outputs using the Adaboost algorithm to produce an effective classification. The objective of this study was to enhance the accuracy of image emotion semantic classification.

## 2. Methods

### 2.1. Low-Level Image Features

The most frequently used low-level visual features of images include colour, texture, and form. An image includes a substantial amount of information; thus, different low-level visual features can produce different psychological reactions. In this study, we employed natural scenery images as examples. Based on the observations of the human visual system and the features of natural scenery images, we determined that colour played an essential role in the understanding of emotion semantics inspired by an image. Conversely, texture and form did not play important roles. Two reasons may explain this difference. The first reason is that the images are irregular, which entails difficulties in extracting texture and form features. The second reason is that the texture and form features do not reflect the emotion semantics of an image in the same manner as colour [11]. Therefore, we extracted colour as the visual feature that best reflected the emotion semantics of a natural scenery image.

### 2.2. Low-Level Colour Feature Extraction and Quantification

#### 2.2.1. Low-Level Colour Feature Extraction

**Figure 1:**
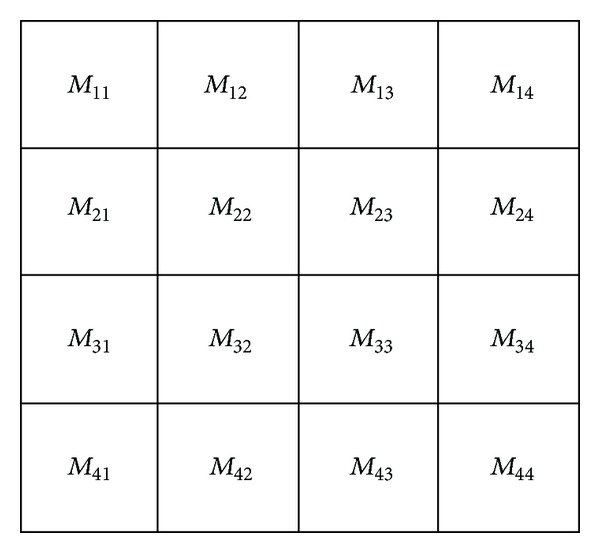
Layout of image blocks.

Image partitioning algorithms are complex to manipulate; moreover, they seldom achieve satisfactory partitioning results [12]. Therefore, we extracted colour characteristics based on deblocking theory [13] and divided an image into 4 × 4 blocks (Figure 1).

The 16 blocks were grouped into three types: *A* = [*M*_11_, *M*_14_, *M*_41_, *M*_44_], *B* = [*M*_12_, *M*_13_, *M*_21_, *M*_24_, *M*_31_, *M*_34_, *M*_42_, *M*_43_], and *C* = [*M*_22_, *M*_23_, *M*_32_, *M*_33_]. As the components of set* C* play the most important role in image understanding, followed by *B* and then *A* [13], we ordered these sets as *C*, *B*, and *A* according to their weights and assigned 3, 2, and 1 to them, respectively. Afterwards, weight number normalisation was performed, and the following results were obtained: *A*, 1/(3 + 2 + 1) = 0.17; *B*, 2/(3 + 2 + 1) = 0.33; and *C*, 3/(3 + 2 + 1) = 0.5. Statistical analyses were performed on the colour histogram of each block. Two colours with the highest number of pixels in the histogram were taken as the low-level colour feature of the block, and the colour characteristics of the whole image were then calculated according to the preset weights.

#### 2.2.2. Colour Space Quantification

Considering that the colour space of hue, saturation, and value (HSV) can adequately reflect human colour cognition, we used HSV as the working space. As the human visual system is more sensitive to hue than to saturation and value, we nonuniformly classified *H* into seven areas and divided saturation and value (SV) into black, grey, and colour areas using the following method from the literature [14].

(1) We subdivided the grey and colour areas. If *v* ∈ [0, 0.2], the area was black. If *s* ∈ [0, 0.2] and *v* ∈ [0.2, 0.8], the area was grey, and *L* = |(*v* − 0.2) × 10| + 1, where *L* symbolises one-dimensional feature vector. If *s* ∈ [0, 0.2] and *v* ∈ (0.8, 1.0], the area was white. If *s* ∈ (0.2, 1.0] and *v* ∈ (0.2, 1.0], a colour area was defined as follows:
  (1) H={0,when  h∈(330,22]1,when  h∈(22,45]2,when  h∈(45,70]3,when  h∈(70,155]4,when  h∈(155,186]5,when  h∈(186,278]6,when  h∈(278,330],S={0,when  s∈(0.2,0.65]1,when  s∈(0.65,1.0],V={0,when  v∈[0,0.2]1,when  v∈(0.2,0.7]2,when  v∈(0.7,1.0].
(2) Colours were quantified according to range and subjective colour perception.
(3) Theoretically, the colour of an object is related to the wavelength and frequency of light. Given that different chromatic lights display different ranges of wavelengths and frequencies, unequal interval quantising of colour hues was performed.
(4) Based on the quantitative levels obtained through the preceding steps, the three components (*H*, *S*, and *V*) were combined according to the following formula: *L* = 4*H* + 2*S* + *V* + 8.

Consequently, 36 colours altogether were obtained. This quantitative method appropriately addressed human vision, reduced colour redundancy, was suitable for grey surfaces, and was easy to calculate. Using this method, the HSV colour space was nonuniformly divided into 10 levels of chromaticity, 2 saturation degrees, and 3 colour hues, which formed a 60-dimensional colour characteristic vector.

### 2.3. Image Semantic Feature Mining

#### 2.3.1. OCC Emotion Model

The OCC model constructs emotional rules by establishing functions to express emotions. *D*(*p*, *e*, *t*) represents the expectation degree of performing event *e* at time *t* for subject *p*. If the expectation produces beneficial results, the function value is positive; otherwise, the function value is negative. Using “anger” as an example, suppose that *I*_*g*_(*p*, *e*, *t*) is the combination of total intensity variables (such as expectation, realisation, and resemblance) and that *P*_*j*_(*p*, *e*, *t*) represents the possibilities of the “anger” state. The rule of the exposure of “anger” is expressed as follows:

 if  *D*(*p*, *e*, *t*) > 0,
 then  *P*_*j*_(*p*, *e*, *t*) = *F*_*j*_(*D*(*p*, *e*, *t*), *I*_*g*_(*p*, *e*, *t*)),

where *F*_*j*_() represents the “anger” function in mathematics. Although this rule does not result in the experience or feeling of anger, it can trigger another rule as follows:

 if  *P*_*j*_(*p*, *e*, *t*) > *T*_*j*_(*p*, *t*);
 then  *I*_*j*_(*p*, *e*, *t*) = *P*_*j*_(*p*, *e*, *t*) − *T*_*j*_(*p*, *t*);
 else  *I*_*j*_(*p*, *e*, *t*) = 0,

where *I*_*j*_ is the “anger” intensity and *T*_*j*_ is a given threshold. This rule activates the “anger” emotion. When the intensity surpasses the assigned threshold value, the emotion “anger” is generated. The intensity value reflects one of the “anger” feelings. For example, “getting angry” corresponds to a moderate value, whereas “hatred” corresponds to a higher value. Other emotional rules were constructed in a similar way.

#### 2.3.2. Analysis of the Emotion Semantics of Colour in Images

In our daily life and work, we generate associations with objects. Similarly, when we view a coloured image, we generate associations with its colour, which is the most direct stimulant; that is, we understand images using human instinctual emotions. For example, we associate the colour orange with golden autumn and fruitful gains. Therefore, it is perceived as a rich and happy colour that symbolises warmth, happiness, and animation. Green is perceived as a nice and graceful colour that is full of vigour and symbolises hope and life.

#### 2.3.3. Semantic Feature Extraction

In this study, we summarised the relations between common colours and basic emotions based on experimental results. First, we downloaded 600 natural scenery images from the Baidu image channel, which is the largest and most renowned search engine in China. Second, we used questionnaires to conduct a survey. The survey subjects included university students and employers; the ages of the subjects ranged from 18 to 45 years. The survey results were statistically analysed.  Table 1 summarises the high-frequency vocabularies and their corresponding relations.

The mapping relationship between colour and human emotion semantics was established using the OCC model. The extracted low-level colour characteristics were then transformed into high-level colour semantic characteristics.

### 2.4. Construction of Adaboost-BP Neural Network Model

**Figure 2:**
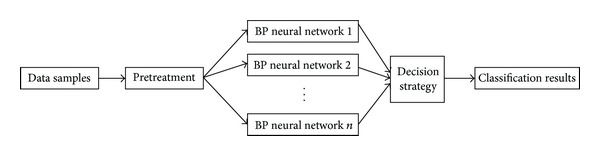
The flow of the algorithm.

The BP neural network is a multilayer feedforward neural network that contains input, hidden, and output layers [15]. It contains the features of forward signal transmission and error counter propagation. It can adjust network weights and thresholds according to predictive errors, enabling the predictive output to continuously approximate the desired output. The Adaboost algorithm is an iterative algorithm. Currently, studies and applications of this algorithm primarily focus on classification [16]. The Adaboost algorithm is capable of increasing the classification accuracy of randomly assigned weak classifiers. By targeting the self-limitations of BP neural networks and the subjective factors of training samples, we established an Adaboost-BP neural network classification model by combining the Adaboost algorithm with BP neural networks. This model employs BP neural networks as weak classifiers. The algorithm scheme is shown in Figure 2.

The algorithm steps are as follows.

(1) Data selection and network initialisation: from the sample space, *m* groups of training data were randomly selected. The distribution weights of the test data were initialised using *D*_*t*_(*i*) = 1/*m*. The neural networks were determined according to the dimensions of the inputs and outputs of the samples. The weights and threshold values of the BP neural networks were initialised.
(2) Weak classifier prediction: when the *t*th weak classifier was trained, the BP neural networks were trained using the training data and subsequently used to predict the output of the training data. The prediction error sum *e*_*t*_ of the prediction sequence *g*(*t*) was calculated based on the following formula:
  (2) $$\begin{array}{c} e_{t}=\underset{i}{\sum }D_{i}\left(i\right)\;i=1,2,\ldots ,m\;\;\left(g\left(t\right)\neq y\right), \end{array}$$

where *g*(*t*) is the prediction classification result and *y* is the expected classification result.

(3) Prediction sequence weight calculation: based on the prediction error *e*_*t*_ of the prediction sequence *g*(*t*), the weight of the sequence *a*_*t*_ was calculated as follows:
  (3) $$\begin{array}{c} a_{t}=\frac{1}{2}\ln\left(\frac{1-e_{t}}{e_{t}}\right). \end{array}$$
(4) Weight adjustment of the test data: according to *a*_*t*_, the weight of the next round of training samples was adjusted. The adjustment formula is expressed as
  (4) $$\begin{array}{c} D_{t+1}\left(i\right)=\frac{D_{t}\left(i\right)}{B_{t}}\times \exp\left[-a_{t}y_{i}g_{t}\left(x_{i}\right)\right],\;i=1,2,\ldots ,m, \end{array}$$

where *B*_*t*_ is the normalising factor that is used to obtain a distribution weight sum of 1 when the weight proportion is constant. *g*_*t*_(*x*_*i*_) is the prediction classification result of *i*th weak classifier.

(5) Strong classification functions: after *T* rounds of training, we obtained *T* groups of weak classification functions *f*(*g*_*t*_, *a*_*t*_). These functions were combined to obtain a strong classification function *h*(*x*) based on the following formula:
  (5) $$\begin{array}{c} h\left(x\right)=\mathrm{sign}\left[\overset{T}{\underset{t=1}{\sum }}a_{t}\cdot f\left(g_{t},a_{t}\right)\right]. \end{array}$$

The constructed model increased or decreased the corresponding weight of a training sample according to fitness after each round of classification and subsequently retrained the weak classifiers using the weighted samples. The training results of these weak classifiers were integrated, and the ultimate outputs were obtained. The rationale of the Adaboost algorithm is to combine the outputs of multiple weak classifiers for effective classification. Therefore, the Adaboost-BP neural network model uses BP neural networks as weak classifiers for repeated training to predict sample outputs. Using the Adaboost algorithm, these BP neural network weak classifiers form a strong classifier.

### 2.5. Image Classification

**Figure 3:**
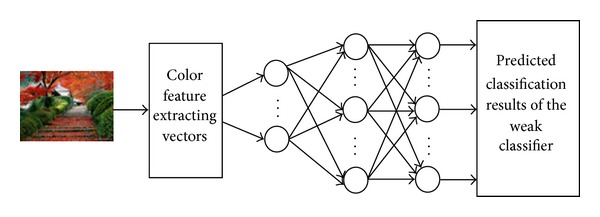
Training process of a BP neural network.

As BP networks possess a simple structure, generalise sufficiently, and are fault-tolerant, self-learning, and self-adapting, we employed them to build the training and learning models for natural image classification. As the input colour feature was 60-dimensional and the images to be classified were subject to 10 classes, we constructed a 60-19-10 network structure. The node number of the hidden layer was obtained based on experimental adjustment. Based on experience, tansig functions were used for the network hidden layer neuron transfer functions, and purelin functions were used for the functions at the output layer. Fifty training steps were used. To increase the generalisation capacity, 300 images were randomly taken from the 500 natural images and used as training samples for each weak classifier. A total of 15 BP neural network weak classifiers with different weights were generated, and they were the same in construction. These weak classifiers were integrated into a powerful classifier based on the algorithm flow in Figure 2 for image classification. The training and learning process of a weak classifier constructed by a BP neural network is illustrated in Figure 3.

## 3. Results

We pretreated the natural scenery images that were downloaded from the Baidu image channel. Images with an unclear style and semantics were removed, and the remaining 600 images were artificially marked. We developed an emotion semantic classification system for natural scenery images using Matlab to perform the training and test processes. In the training stage, we selected 500 images, which were subject to different styles, from the original set of 600 images and assigned them to the training set. In the test stage, we assigned the remaining 100 images to the test set.

**Figure 4:**
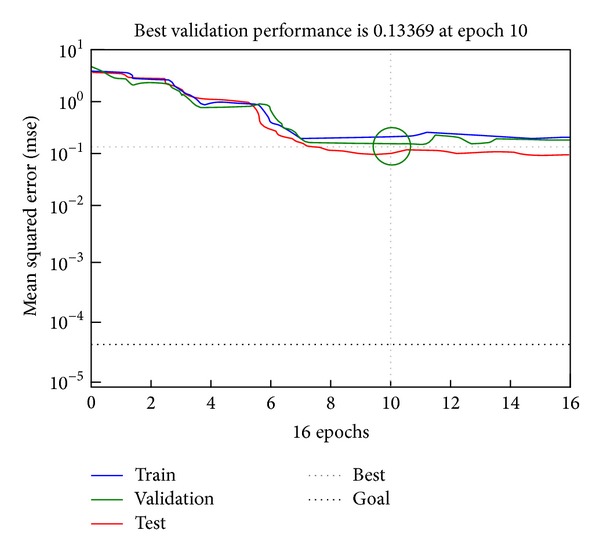
Training process of the Adaboost-BP neutral network model.

After the network model was established, we selected a certain number of training samples for network training. To obtain a strong classification capacity for the network, we adjusted the node number of the hidden layer for each classifier. In this study, we selected 500 images, which were subject to various classes, as the training samples and retained the remaining 100 images as test samples. The desired output vector was denoted by *W* = {*w*_1_, *w*_2_,…, *w*_10_}, where *w*_*i*_ is the image category. The training results of the Adaboost-BP neural network model are shown in Figure 4.

**Figure 5:**
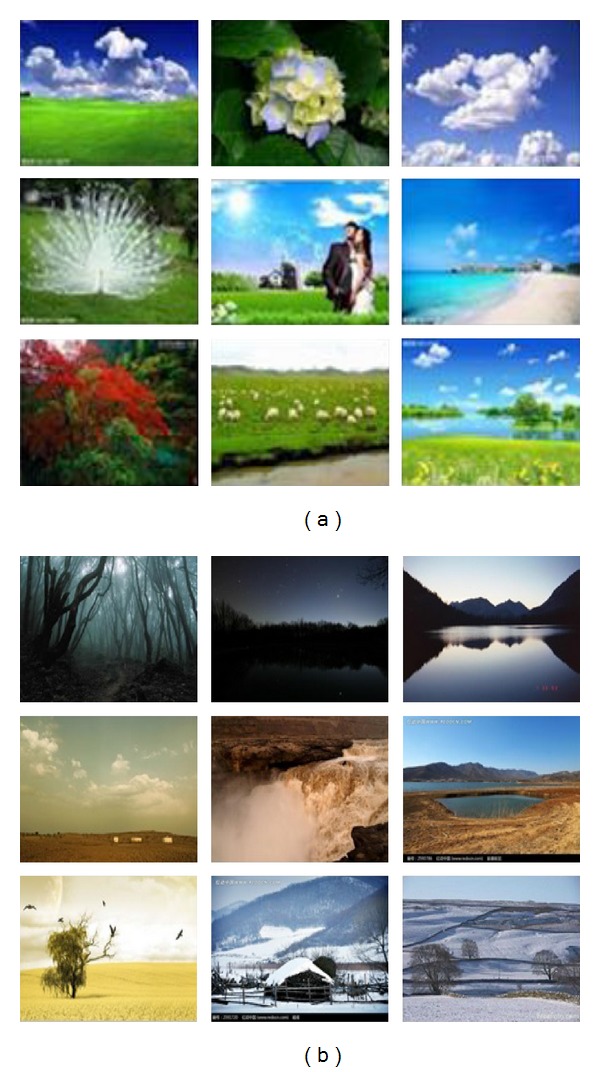
Partial retrieval results for the emotional words “hopeful” and “frustrating,” respectively. (a) Hopeful. (b) Frustrating.

Figures 5(a) and 5(b) show the results retrieved by the system for the emotional word “hopeful” and “frustrating,” respectively.

**Figure 6:**
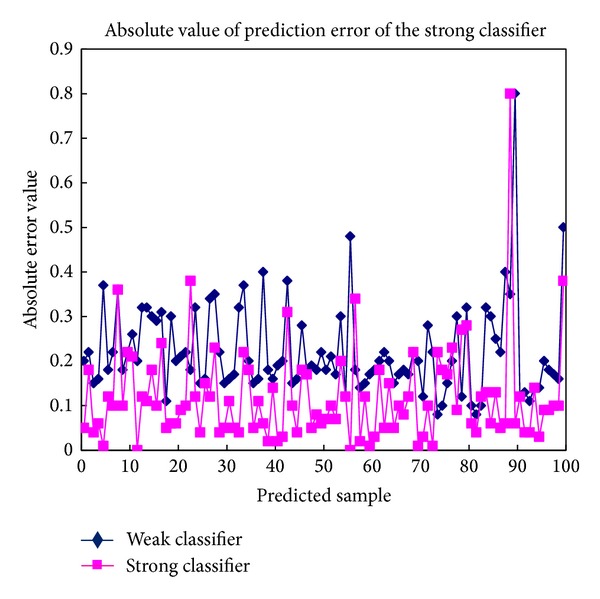
Absolute values of the predicted classification errors of the Adaboost-BP neural network model.

Figure 6 shows the absolute values of the predicted classification errors of the Adaboost-BP neural network model. As shown in this figure, the predicted classification errors of the strong classifiers were smaller than the predicted classification errors of the weak classifiers.

Recall and precision rates are two important indices for evaluating the performance of image classification systems. The recall rate reflects the ability of a system to classify relevant images based on the following formula: recall rate = the number of retrieved relevant images/the number of all relevant images in the retrieval system × 100%. The precision rate reflects the ability to reject irrelevant images based on the following formula: precision rate = the number of the retrieved relevant images/the number of retrieved images × 100%.

Higher precision and recall rates indicate adequate classification by the system. However, the majority of systems cannot simultaneously obtain the maximum values of both rates, namely, a high precision rate with a low recall rate or vice versa. All systems should frequently optimise these two indices. To verify the efficiency of the classification algorithm of the Adaboost-BP neural network model, we randomly extracted 350 images that contained various emotions. The recall and accuracy rates were calculated and compared with the results obtained using conventional BP neural networks. The results are summarised in Table 2.

**Figure 7:**
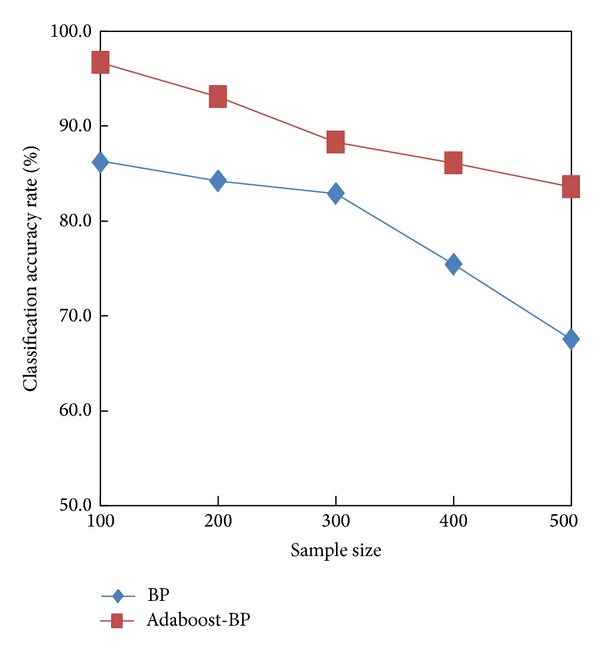
Comparison of the average classification accuracy rates of the BP neural network model and the Adaboost-BP neural network model.

To further validate the effectiveness of the algorithm used in this study, we calculated the average classification accuracy rates of the Adaboost-BP and BP models using different sizes of test samples, and the results are shown in Figure 7. As shown in Table 2 and Figure 7, the classification recall rate, accuracy rate, and average accuracy rate of the Adaboost-BP algorithm were higher than those of the BP model. Furthermore, although the average accuracy rates of both models decreased as the sample size increased, the accuracy rate of BP decreased rapidly after the sample size exceeded 300, whereas the accuracy rate of Adaboost-BP displayed a gradual decrease.

## 4. Discussion

In this study, we established an emotion space using the OCC emotion model and explored the relationship between natural scenery images and human image semantic understanding. Based on the Adaboost algorithm, we utilised BP neural networks as weak classifiers and combined their outputs based on the Adaboost algorithm to generate effective classification. According to the results of the simulation experiment, our methods possessed significant advantages in emotion comprehension, which presents problems with high subjectivity and uncertainty. It effectively increased the accuracy rate of image emotional semantic classification.

Colour plays an essential role in understanding the emotion semantics inspired by an image. When we view a coloured image, we generate associations with its colour. To start this study, we explored the relations between common colours and basic emotions based on questionnaires. Figures 5(a) and 5(b) showed that colour is the most emotional among all the visual features contained in images and that conspicuous coloured blocks can attract users' attention immediately and induce subjective perceptions. Colours with different degrees of saturation lead to different perceptions. High-level purity possesses a powerful impact force that helps produce a strong visual perception, whereas low-level purity induces a soft, simple, and unadorned perception. In terms of brightness, bright colours produce a relaxed perception, whereas dark colours leave a sense of heaviness. In terms of hue, warm colours have diffusibility, whereas cool colours have contractility. For instance, red, yellow, and orange (warm colours) give rise to perceptions of warmth, ardour, and happiness, whereas blue, green, and white (cool colours) give the perceptions of serenity, freshness, and sternness. Red symbolises ardour, happiness, activeness, and passion, which induces excited and energetic perceptions. Orange symbolises vigour and warmth. Yellow symbolises brightness, hope, and wisdom. Green symbolises peace and viridity, inducing affectionate, peaceful, and pleasant perceptions. Blue symbolises tranquillity, wisdom, and profoundness, inducing pure, elegant, and fresh perceptions. Purple symbolises luxury. White symbolises purity, nobility, and frankness, inducing lucid, lively, and quiet feelings. Black symbolises gravity, solemn, horror, and sorrow, inducing perceptions of solemn silence and mystery.

From the perspective of emotional semantic understanding, we established a model and classification categories for a customer's subjective understanding of images and proposed new classification methods for image emotional semantics. The majority of previous studies employed one machine learning method or combined two methods for classification. Given that image semantics are difficult to evaluate, these methods frequently produce low accuracy rates. In contrast, we constructed a powerful classifier based on the classification of weak classifiers. The results of the simulation experiment showed that the Adaboost-BP neural network algorithm attained mean recall and precision rates of 91.5% and 86.7%, respectively, for natural scenery semantic classification, which show an increase of 3.5% and 4.2%, respectively, compared with the mean recall and precision rates of the BP neural network classification algorithm (88.0% and 82.5%, resp.). The Adaboost algorithm showed superiority in constructing a strong classifier, and it solved the existing problems in the BP neural network, such as slow convergence speed and poor generalisation ability. Machajdik and Hanbury extracted image features from the perspectives of psychology and arts and proposed a method for semantic classification [17]. They extracted and combined low-level features that represented the emotional semantic of an image, then used these features for image semantic classification of international affective pictures (IAPs) and abstract paintings, and obtained recall and precision rates of 65% and 68%, respectively. Our method significantly exceeded these values (by approximately 15%) for two reasons. First, the powerful classifier constructed in this study exhibited a superior classification effect. Second, we used natural scenery images to test the classification effect of the Adaboost-BP method. Semantics implicated by natural scenery images are more discernible than semantics implicated by IAPs and abstract paintings, which may be partially responsible for these differences.

This study provides not only a new method for image emotional semantic understanding but also a new idea for classifier design in other fields.

This study presented some limitations. First, the evaluation of the neural network model requires a large number of training sets. The more training data provided, the more accurate the test results. However, this process will consume a considerable amount of time and human resources. Obtaining a high classification rate based on a small number of sample sets will be the focus of future research. Second, the semantics implicated by different types of images are abundant and complex. The development of semantic understanding models, the standardisation of categories of image emotions, and improvements in the precision and retrieval efficiency of image classification should also be addressed by future studies.

## 5. Conclusions

The design of computers that are able to recognise and express emotions as human beings do and create harmonious human-computer interactions represents an important challenge. This study explored the relationship between images of natural scenery and the human comprehension of image semantics using the OCC emotion model. A strong classifier was constructed by integrating the Adaboost algorithm and the BP neural network method, which resulted in the automatic emotion classification of natural scenery images. The method proposed in this study possesses advantages for understanding subjective and highly uncertain emotions and achieved acceptable results.

However, neural network models require large numbers of training sets. Although more training sets produce more accurate test results, a considerable amount of time and human resources are consumed during the process. Therefore, methods to acquire a high marking accuracy based on a small number of training sets will be the focus of future studies. As the semantics implicated by natural scenery images are abundant, a method to reasonably standardise image emotion categories remains unexplored.

## Figures and Tables

**Table 1:** A comparison of colour and emotion semantics.

Colour	Semantic description	OCC emotional word(s)
Red	Ardent, happy, passionate, and romantic	Happy, proud
Orange	Warm, friendly, and gentle	Happy
Yellow	Bright, mild, and active	Happy, relaxing
Green	Hopeful, living, full of vigour, and fresh	Hopeful
Cyan	Youthful, pretty	Hopeful, relaxing
Blue	Neat, calm, cold, indifferent, and sorrowful	Sorrowful
Purple	Romantic, graceful, mysterious, and noble	Proud
White	Monotonous, indifferent, and poor	Frustrating
Grey	Casual, old, and indifferent	Frustrating, dreadful
Black	Solemn, related to death, serious, and horrific	Angry, dreadful, and disgusting

**Table 2:** Comparison of the experimental results with the results obtained using the BP and Adaboost-BP methods.

Emotion type	Recall rate of BP (%)	Recall rate of Adaboost-BP (%)	Precision rate of BP (%)	Precision rate of Adaboost-BP (%)
Sorrowful	89.7%	93.5%	86.4%	88.9%
Frightened	88.3%	90.8%	82.6%	86.4%
Disgusted	82.9%	87.2%	73.1%	79.8%
Relaxed	91.6%	93.2%	88.5%	90.2%
Angry	90.1%	93.1%	84.9%	89.5%
Frustrated	79.3%	86.2%	71.6%	77.8%
Scared	90.9%	93.6%	85.7%	90.1%
Happy	93.6%	95.8%	89.1%	92.2%
Proud	83.0%	87.1%	78.3%	84.3%
Hopeful	91.4%	93.8%	85.7%	89.6%
